# The lived experience of people with diabetes using off-the-shelf prescription footwear in Singapore: a qualitative study using interpretative phenomenological analysis

**DOI:** 10.1186/s13047-019-0329-y

**Published:** 2019-03-21

**Authors:** Sheena Tan, Hazel Horobin, Thanaporn Tunprasert

**Affiliations:** 1 Podiatry Department, Ng Teng Fong General Hospital, 1 Jurong East Street 21, Singapore, 609606 Singapore; 2 University of Brighton, School of Health Professions, 49 Darley Road, Eastbourne, BN20 7UR UK; 3 University of Brighton, School of Health Professions, 49 Darley Road, Eastbourne, BN20 7UR UK

**Keywords:** Footwear, Lived-experience, Interpretative phenomenological analysis, Adherence, Singapore, Ulceration, Diabetes

## Abstract

**Background:**

Diabetic foot ulceration (DFUs) is increasingly prevalent in Singapore. Appropriate management is important since DFU brings with it an associated risk for lower limb amputations, high morbidity rates and costs. Footwear prescription has been a part of clinical guidelines to manage DFUs. However, adherence to prescription footwear is typically poor amongst patients. Reasons for this have been explored in Northern American and Western European studies, but not in Singapore’s context. As cultural and climate differences limit transferability of findings from existing studies to individuals in Singapore, this study aims to explore the lived experiences of participants with diabetes using prescription footwear in Singapore.

**Methods:**

This was a qualitative study using interpretative phenomenological analysis (IPA) to understand some people’s personal experience of using off the shelf prescription footwear. A total of 8 people with diabetes who received prescription footwear as part of their diabetic foot management were recruited. All participants provided written consent and took part in a semi-structured interview lasting up to an hour. Interviews were digitally recorded, transcribed and analysed using an IPA approach.

**Findings and discussion:**

The analysis identified the super-ordinate themes of 1) security and 2) acceptance with sub-themes of 1.1) risk and 1.2) protection and 2.1) personal and social acceptance and 2.2) social and cultural acceptance respectively that inter-related to influence how participants’ made footwear decisions. This process of evaluation was portrayed to be a fluctuant one, making it difficult to predict yet necessary to understand. A modified seesaw model of adherence is suggested to explain this decision-making process.

**Conclusions:**

The complex manner by which participants grappled with security and acceptance is often overlooked when footwear is prescribed, highlighting a need for a more collaborative clinician-patient partnership for these issues to surface in clinical practice. Furthermore, prescription footwear should be seen more holistically. Empowering patients with choice to select from a range of therapeutic yet normalised footwear could increase the level of security and acceptance they experience with its use.

## Background

Diabetic foot ulcerations (DFUs) affect a significant number of patients with implications on morbidity, quality of life, costs and amputation risk [1–4]. Similarly in Singapore, DFUs are significant risk factors for lower limb amputations, leading to high morbidity rates and costs [5]. However, it has been suggested that at least 40% of such amputations can be prevented with appropriate management of the diabetic foot [6]. Amidst a 33% increase in diabetes-related lower limb amputations over the past decade in Singapore, the need for appropriate management of the diabetic foot has never been more apparent [7].

Trauma from footwear has been identified as the leading precursor to foot ulceration in diabetic patients [6, 8–11]. Recognizing this, appropriate footwear prescription has been part of numerous national and international guidelines on managing the diabetic foot [12–14]. However, evidence on its efficacy in preventing and managing DFUs has been inconclusive. While use of prescription footwear has been effective in reducing plantar pressures [15–20], this has not translated into a reduction in ulceration rates. This discrepancy has been largely attributed to patients’ poor adherence to prescription footwear [21–23]. While it is suggested that prescription footwear should be worn 60–80% of the day for significant reduction in ulceration risks [24, 25], studies suggest that patients only use prescription footwear 22–36% of the time [21, 26, 27].

Variations in quantitative data [21, 23, 26] on factors affecting patient adherence to prescription footwear suggest that general predictors of the whole group may not exist [28]. Consequently, qualitative studies that sought an in-depth understanding on patients’ perspectives were employed to understand this behaviour [29]. Differences in perspectives between people with diabetes and healthcare professionals on prescription footwear were identified [30]. The authors found that healthcare professionals focused more on limiting morbidity with prescription footwear, while people with diabetes understood its therapeutic purpose but processed its use in the context of their daily lives [30]. They identified a knowledge-action gap in the use of prescription of footwear amongst people with diabetes, where the process of accommodating prescription footwear in their daily lives was seldom addressed.

Another study explored the lived-experiences of people with diabetic neuropathy and prescription footwear to understand the unique perspectives associated with the use of said footwear [31]. It revealed how adherence is a process where individuals gradually shift their priorities over time to eventually result in higher adherence to prescription footwear. This study highlighted the necessary adjustment of personal values when people with diabetes chose to use prescription footwear [31].

While these studies have shed light on the experiences of people with diabetes using prescription footwear in Western Europe [30, 31], its transferability is limited in Singapore’s context. As socio-cultural and climate differences have been known to influence footwear habits [32–34], experiences of people with diabetes using prescription footwear in Singapore are likely to be different from those in North America and Western Europe.

To date, little is understood about the experiences of people with diabetes using prescription footwear in Singapore although it is used regularly in clinical practice [35]. This research aims to explore the personal experiences of participants with diabetes using prescription footwear, so as to understand how footwear decisions are made and influenced in the Singaporean population. Findings from this research may also aid clinicians in providing adequate support to patients when prescribing footwear.

## Methods

Interpretative phenomenological analysis (IPA) was used to analyse data from this study. This methodology was chosen as it posits that lived-experiences can be understood via examination and interpretation of meanings that people impress on it [36]. As such, it is well positioned to understand participants’ personal experience of using prescription footwear in their daily lives.

### Ethics

Ethics approval was obtained from the University of Brighton’s Faculty of Health Research Ethics and Governance Committee (FHREGC) and from the National Healthcare Group Domain Specific Review Board (NHGDSRB) in Singapore.

### Inclusion and exclusion criteria

People with diabetes who are ambulatory, have been prescribed off-the-shelf prescription footwear and have the ability to consent were included in the study. Those who are not able to converse in English, are using temporary wound sandals or are unable to take part in an interview lasting up to 60 min were excluded.

### Recruitment

Posters and brochures containing research information were made available at Podiatry clinics in Ng Teng Fong General Hospital, Singapore. Interested volunteers who met the inclusion and exclusion criteria were encouraged by their clinicians to contact the researcher via email or a phone call for more information. The researcher responded to phone calls and emails from potential participants within 2 working days to provide more information and address queries. Once the participant volunteered to take part in the study, a date and time for an interview was arranged at his convenience. There was a 24-h cooling-off period between all emails received by the researcher and responses sent to the volunteers to minimise the likelihood of coercion.

A total of 8 participants (7 males and 1 female) were recruited in this process. This is deemed an appropriate number for research of this nature [36].

### Consent

The principal researcher verbally went through the contents of the information sheet and consent form and all questions were answered prior to the volunteer’s signing and commencement of the interview. Participants were also informed that the data they contribute to the study would be used for research publications and conference presentations, and that pseudonyms would replace their original names in these publications.

### Interviews

Individual semi-structured interviews, a preferred method for data collection in IPA were conducted and digitally recorded [37]. This method allowed participants the space to reflect, speak and be heard, facilitating more in-depth discussion [36].

The researcher’s role as a podiatrist at the hospital where the study was conducted was fully disclosed to participants. Although the researcher was not actively involved in the participants’ care during the duration of the study, the researcher had previously treated and interacted with all participants. This was beneficial as an existing relationship with participants provided a platform for rapport building during the interview. However, the researcher was also aware that her pre-conceived notions about the therapeutic benefits of prescription footwear could potentially bias data collection and interpretation. As such, a reflexive journal was used during this process to bracket and suspend her personal assumptions and emotions, so as to ensure minimal bias on the data collection and interpretation process. The researcher also addressed her role at the start of each interview to ensure that participants understood her role as a researcher, and not a podiatrist, during the interview. The interviews were conducted in a casual manner in a room away from the podiatry clinics to reinforce this segregation of roles. All interviews lasted up to an hour.

The interview schedule was designed to explore the impact on prescription footwear on the daily lives of participants and their perspectives on its use. Taking reference from that used by Paton and colleagues in a similar study [31], a pilot study was conducted with two participants. However, participants were observed to be unfamiliar with responding to broad-topic questions and elaborating on their experience. As such, the interview schedule was modified to include more specific questions that allowed the interviewer to build context about participants’ daily lives and explore the participants’ experience in a more directed manner. The order of questions was used flexibly depending on the flow of the interview. The interview schedule covered:

- Daily footwear routine and the reasoning behind it
- Perceived impact of prescription footwear on daily life
- Perceived impact of prescription footwear on identity
- Wearing prescription footwear in social settings
- Additional factors influencing footwear decision

### Analysis

Analysis of data involved six stages of IPA and was followed meticulously throughout [36]. It began with close reading of the transcript to allow an overall structure to develop while the researcher’s recollections and emotions regarding the interview were bracketed off in a reflexive journal. This was done to reduce the influence of personal bias, prior knowledge and pre-conceived notions during the analysis.

Subsequently, exploratory notes on observations, context and language used were noted on a copy of the transcript. After which descriptive, linguistic and conceptual comments were noted [36]. Emergent themes were mapped based on interrelations of exploratory notes, reflecting the participant’s words and thoughts, as well as the analyst’s interpretation [36].

Themes for the whole transcript were compiled while connections between emergent themes were identified. These themes were grouped according to conceptual similarities to highlight important aspects of the participant’s account [38]. This process was repeated for each transcript on its own terms, keeping with IPA’s idiographic commitment [36]. Super-ordinate themes were then developed at a higher level of abstraction based on emergent themes across transcripts.

## Results

The analysis identified two superordinate themes: 1) security and 2) acceptance that interrelate with each other to influence footwear choices amongst participants in Singapore. Under the theme of security are subthemes of 1.1) protection and 1.2) risk; while under the theme of acceptance are subthemes of 2.1) personal and social acceptance and 2.2) social and cultural acceptance Table 1

**Table 1:**
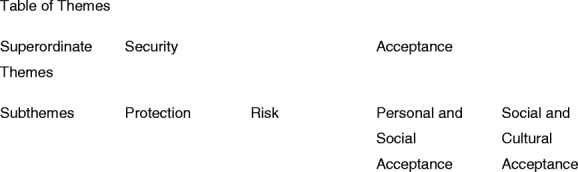
Table of Themes

.

These themes revealed a more pragmatic rather than emotive slant on participants’ experiences and may have been influenced by the type of interaction that both the participants and the researcher were more inclined to. This may be partially attributed to the Asian cultural norm where emotions are often not accorded sufficient importance and play a less significant role in decisions. Therefore, while the emotional experience of living with prescription footwear was explored, cultural predispositions influencing both participants and the researcher likely influenced the emphasis on pragmatic implications in the experience of using prescription footwear [39], resulting in the following superordinate themes and subthemes.

This section will explore the experience of 8 participants linked by the common experience of being prescribed off-the-shelf prescription footwear.

### Superordinate theme 1. Security (protection; risk)

Security is defined as ‘the state of feeling safe, stable and free from fear or anxiety’ [40]. Participants revealed that they commonly sought after a sense of security when using prescription footwear and the degree of security they experienced depended on their evaluation of protection and risk derived from using prescription footwear in that situation.

#### Subtheme 1.1 protection

As evident in similar studies, participants in this study were aware of the risk of injuries and the need for protection of their feet [30, 31]. There was unanimous acknowledgement of the importance of protection offered by prescription footwear amongst participants interviewed.

*“Oh that one is for leisuring shoe. This one is for taking care of my foot.” [Dan interview transcript]*

*“I feel like it…is better than those ordinary shoe. It’s VERY good… better. Is it…something is protect…I think one thing is like a a child protect by the her mother or what like that. That’s why you wear already right, it’s very comfortable very.” [Sarah interview transcript]*

However, for some, the level of protection offered by prescription footwear seemed uncertain. While they say similar things, a lack of conviction leaves one wondering if this need was genuinely felt or if it is a superficial repetition of what they have been told.

*“Er… I reckon it’s to yeah help my legs er through this diabetic er what d’you call it the veins are thinning and all that so I’m feeling quite uncomfortable so it’s there to help yeah preserve my legs further lah… from any injury.” [Peter interview transcript]*

*“Mmm, actually… it was fine because it does help my feet. Ah it’s comfortable and then the insoles is helping to reduce the risk of getting a wound.” [Michael interview transcript]*

*“It’s, it’s about safety conscience towards me because when I wear this I know this is a shoe that will prevent a lot of things for me…right?... Mmm. Preventing from getting hurt… Y’know… And preventing from getting more er, more pain or whatever on the leg. So, this shoes is doing a good job.” [John interview transcript]*

Contrastingly, this need for protection is portrayed as a significant priority for other participants and had a direct influence on their footwear choice.

*“Cos a lot of people maybe they were forced to buy they will you have to use it know, for me is I want to use it so that my leg’s comfortable. And prevent injury lah.” [Lawrence interview transcript]*

*“Because because I want safe, I want comfort, it’s this shoe. If I wear another shoe, it means I’m not comfortable with it and then there’s no safety, no point using lah. Ah. So the priority now is to save my feet. With wearing these these shoe. And I feel that this is one is the best so far.” [James interview transcript]*

Therefore, despite the often-assumed safe nature of prescription footwear, participants’ experiences suggest that the concept of security is an uncertain one that is constantly being re-evaluated against situational factors.

#### Subtheme 1.2 risk

While prescription footwear is often prescribed to protect participants’ feet, it does not necessarily invoke a sense of security. It was surprising to note that majority of participants perceived a risk of injury with its use. This was confusing for participants as they tried to reconcile its protective purpose with the every day risks they encounter.

*“(This shoe) is a bit wobbly you know? There was once I, I was walking across a car park then I didn’t see the there’s a metal thing coming out on the road then I tripped there on (this shoe) I fell down. Yeah but I didn’t know (tsk) because this is a bit wobbly you know? In a way it’s wobbly, I don’t know why.” [Lawrence interview transcript]*

*“But this one, originally I had problems because of this shoe because we were when I was travelling and all that maybe I was brushing against these things ah. In the plane or whatever. So when I came back, y’know, it started having wounds.” [Peter interview transcript]*

The risk involved with using prescription footwear in wet weather in Singapore was most significant to participants. As the off-the-shelf prescription footwear uppers are made with a non-waterproof elastic polyurethane fabric, participants expressed the extent of getting prescription footwear wet in wet weather with severity, revealing the significance of the problem.

*“Because this one cannot wear when it’s raining then you hav you ha… it’s going to get flooded… Rainy. Because this one is once is rains, some places you walk, the water just keep coming in lor.” [Lawrence interview transcript]*

Participants associated the risk of getting prescription footwear wet with a possible deterioration of their foot condition, especially in those with existing ulceration and employed various means to keep their feet dry.

*“So I also never walk in the rain lah. Right now I try to avoid all those things lah. Because I scared this one got wet and then I got the only pair I have wet then I cannot go out you see? (laughs)…Ah, try to walk shelter and then ah, if no choice then have to walk have to walk lah. But then check lah, scared wet ah, then you wear then after that er leg got problem.” [James interview transcript]*

*“But of course I try not to (wear these shoes), because the shoes are really like (tsk) plain cloth shoes and then you know, if you fall down, it’ll tear and then it’ll expose your skin your…and then you know if it’s raining then probably, definitely going to get wet (laughs)…right? So… and then if you have a wound, you got to keep it dry yeah.” [Michael interview transcript]*

*“Here right rain comes I have to stand somewhere else until the rain stop. Because I’m afraid, because I’m afraid it might get wet. You know? And all this kind. That’s the only thing lah. So usually what I do is I carry plastic bag. So when it rains (chuckles), I cover. I cover then I walk. As I said, when you wear the plastic bag and walk it is very dangerous. Yeah it’s very dangerous. So with that say I walk slow” [John interview transcript]*

However, attempts to reduce the risk of getting their footwear wet perpetuated other health risks such as limited physical activity and inappropriate footwear use. Furthermore, it was revealed that the risks involved with using prescription footwear in wet weather were not addressed in clinic, resulting in fear about using prescription footwear.

*“Actually first time when I wear this shoe ah [CT] I was quite scared, I completely see water only I completely refuse to walk that path with this shoe. Yeah. Because I don’t know anything about this shoe. It does not say anything here. I look at the sole, it’s like ordinary rubber or anything like that. So I keep thinking wah lao eh (Hokkien exclamation used to express exasperation) this shoe, wet how?” [Adam interview transcript]*

Weighing the risks involved with using the very footwear meant to protect them was a difficult contradiction to reconcile. Following up with Michael on the risks he experienced, he was asked if he felt unsafe in prescription footwear, to which he replied:

*“Actually not. Actually it’s ok. The shoes are ok. But it’s just that yeah sometimes erm you know you just might want to wear proper shoes for proper activities that you want to do lah.” [Michael interview transcript]*

Like Michael, this contradiction kept participants’ perception of prescription footwear in a state of flux, re-weighing the risks and benefits of its use according to different situations, often having to come to a compromise.

### Superordinate theme 2. Acceptance (personal and social; cultural and social)

Participants described a process of acceptance of prescription footwear on a personal, social and cultural level. While these were individual processes at times, they interacted often to influence one another. Social acceptance was revealed to be a running thread holding these different facets of acceptance together, highlighting the importance of addressing the social impact of using prescription footwear.

#### Subtheme 2.1 personal and social acceptance

Personal acceptance was revealed to be a process of adapting personal values regarding footwear. Participants described a process of getting used to their new image and rationalizing its functional benefits.

*“Well, the shoes, is at first I said I’m funny then after that I get, when I get used to it I think now I’m, I walk without the sh… I have to walk with the shoes… because I ca…you know…a friend. Like a best friend you know… walking around with you… So erm, I said is the shoes is safe, comfortable…ok? And er what do you call that…and and it gives you confidence in walking.” [John interview transcript]*

*“Ok ah… anyone seeing this wearing this shoe they know that er I’m wearing this because I’m having some issues with the legs you see...But that that doesn’t bother me.” [Peter interview transcript]*

*“Actually I know it’s like what people thought it’s like very bulky lah but I don’t think so but at it is fit me, very comfortable, not tight, not everything.” [Sarah interview transcript]*

Tenets of social acceptance can either reinforce the process of adapting one’s personal values to accept prescription footwear, or reject it.

*“Er…I just ignore it and you know people understand understand. Most of the people who meet me and see me, they understand my condition you see. So I feel eh nothing wrong with the shoe what. It’s nothing. It it yeah ah even I think we can we can see some more awful shoes that yeah girls wear. I I I hari raya the other time, can see ah some so awful also they wear. I my one also not so bad lah.” [James interview transcript]*

*“Sometimes if you go for certain function, need to wear those type of shoe. Yeah you don’t expect me to wear this one to go for some high-class thing you know (laughs)? You know you know? Because it looks very funny you know?” [Lawrence interview transcript]*

The consideration of social acceptance appears more significant in the lives of participants who are currently working, as they engaged in business meetings demanding certain dress etiquettes. Prescription footwear was perceived as socially unacceptable in those circumstances, resulting in participants rejecting its use.

*“(laughs softly) Yeah like I said lah, probably like you know you don’t want to go for meetings or weddings or something like that in these kind of shoes lah… Well because it’s doesn’t look nice (laughs). Yeah. So no choice lah. You have to wear other shoes.” [Michael interview transcript]*

*“Because we, I mean sometimes we have we have certain things like er, for example if we have those, we go and arrange Donald Trump those meeting and you go there you wear this kind of shoe then you’ll be trouble right?” [Lawrence interview transcript]*

This rejection was also evident, to a lesser extent, in the lives of other participants when they engaged in functions where prescription footwear was deemed socially inappropriate.

*“Wearing these shoes doesn’t suit. Sometimes wedding you know you wear a three-piece suit and then you go and wear this kind of shoes? People will look at it and it’s funny.” [John interview transcript]*

*“‘Like erm…because now they got no pattern nothing you see just black colour. So need some that’s just a what ah need some pattern ah all that. That means you can go if if if is is make like that ah, you can wear the shoe go go party goooo wedding go anything.” [Sarah interview transcript]*

Participants associated socially acceptable footwear with an appearance of normalcy. Conversely, the use of prescription footwear was viewed as socially unacceptable as it was perceived to look inferior. When asked how he would prefer prescription footwear to look like, James revealed the following:

*“More sporty lah maybe. Yeah. So it doesn’t looks so inferior with others you see. So i-i-if if I come that’s why I say when I come for appointment ah it’s nothing you see, but you go walk on the street, your shoes definitely is totally different from others ah.” [James interview transcript]*

This highlighted a need for social conformability in one’s acceptance of prescription footwear.

#### Subtheme 2.2 cultural and social acceptance

The interaction of cultural and social acceptance was significant in regulating use of prescription footwear at home. Low use of footwear at home in this study appeared to be motivated by deep-seated socio-cultural norms, where footwear use at home is seen as unacceptable.

*“When I go out I have to wear. At home only at home I don’t wear. At home I wear the sole only… Ley chey (colloquial term for troublesome) lah. Whatever way, I’m not used to wearing footwear at home what.” [Adam interview transcript]*

*“Cos it’s also, the norm we normally don’t wear outdoor and indoor right?.” [Lawrence interview transcript]*

*“Because in a malay custom, they never wear shoes in the house…And including me. Because of what, I was been trained by both of my parents.” [Dan interview transcript]*

*“Ah because (tsk) you know lah, we walk everywhere dirty lah to go inside the house… Maybe (tsk) not for us lah. Like Malay people lah, don’t don’t use, I think Chinese also now also never wear the shoe inside lah wear slipper lah is one home shoe right? So because of that lah, and then I only I only got one pair. So, normally I take out this shoe lah.” [James interview transcript]*

As a result of these cultural norms, use of prescription footwear at home is rejected straightaway and participants do not even engage in a consideration of risks or comfort with its use. While there is occasional allowance for deviation, it appears that any deviation from these cultural norms required gaining social approval from friends and relatives prior to its use.

*“Because most relatives or friends they know about my situation so they are ok with it. So I usually bring a extra pair of slippers in my bag. Clean ones, so that I can walk in the house. You know, not those that I’ve been walking everywhere.” [Michael interview transcript]*

Even then, there is an emphasis on not using the same pair of shoes outdoors and indoors, resulting in participants using alternative footwear at home instead of prescription footwear.

## Discussion

### Security (protection)

Healthcare professionals often view the use of prescription footwear as a mean to limit morbidity [30]. As a result, it is unsurprising that the patient’s acknowledgement of the need for protection from injuries is often seen as a successful endpoint to the intervention. Yet, studies have also shown that patient education has limited effectiveness on the use of prescription footwear [41, 42] and there is growing acknowledgement of the knowledge-action gap that exists as patients process the use of prescription footwear in their daily lives [30, 31]. While protection offered by prescription footwear may be apparent to patients and professionals alike, varying levels of conviction regarding its need appears to contribute to this knowledge-action gap that determines patients’ behaviours.

Vileikyte and colleagues’ model of adherence to foot care suggests that tangible experiences of developing a foot ulcer, rather than theoretical ideals about preventing it, elicit a stronger emotional response to motivate behaviour change [43]. While this may explain why some participants, who had histories of DFUs, portrayed the need for protection as a priority, it does not explain the differing levels of convictions on the protective effect of prescription footwear, as those who lacked conviction on its protectiveness had a history of foot ulceration too. Instead, this study suggests that there is unaddressed contradiction between what some participants have been told and what they truly believe despite their history of ulceration, revealing an underlying struggle to accept the protective value of prescription footwear.

Their unwillingness to reveal these contradictory beliefs during the interview hints at a paternalistic model of healthcare where physicians are assumed to know better about what is good for them [44]. Therefore, instead of voicing their opinions, participants took on a superficial acceptance of the protective nature of prescription footwear. It is necessary for clinicians to pay attention to these nuanced differences in patients’ perspectives when prescribing footwear, as it is easy to take the patients’ acknowledgement of prescription footwear’s protective features as a successful endpoint. To do so would require a shift from a professional-centric approach to footwear prescription to a more collaborative clinician-patient relationship, where the patient is included in a sharing his view and has a say in the decision-making process [30].

It is also worth noting that protection is closely linked to the concept of comfort as participants interchangeably used one to mean the other. While protection is seen as a more abstract concept, physical comfort reinforces its presence, encouraging continuation of its use. This echoes Kolcaba’s comfort theory, which suggests addressing patients’ needs by enhancing their comfort levels, which in turn enhances continuation of health-seeking behaviours [45].

Previous studies have suggested that footwear aesthetics is a more significant priority than comfort amongst people with diabetes [26, 45]. A possible reason for this is that participants in these studies were asked to select their preferences based on appearance of a range of footwear and not actual usage [46]. Furthermore, they may have prioritized aesthetics over comfort due to their lower satisfaction in prescription footwear aesthetics (Visual analogue scale (VAS) = 6.8) as compared to their satisfaction in prescription footwear’s comfort (VAS = 8.1) [26]. Therefore, while comfort has been portrayed as a seemingly lower priority in footwear requirements amongst people with diabetes, its close association with a sense of protection suggests it remained an important feature to participants.

### Security (risks)

Previous studies suggested that perception of risks influences health behaviours and that patients weighed risks of foot ulceration against risks involved with appearing normal in regulating footwear choices [31, 47]. These studies depicted trade-offs in appearance for the therapeutic benefits of health devices as they were based on the assumption that such devices reduced injury risks. However, participants in this study challenged this assumption, revealing risks of injuries associated with prescription footwear use, especially in wet weather. The frequency of getting caught in heavy downpours likely contributes to the significance of this risk perceived by participants in Singapore, as compared to participants in similar studies elsewhere [31].

This contradiction kept participants’ perception of prescription footwear in a state of flux and they re-weighed the risks and benefits of its use according to different situations, often having to come to a compromise. It therefore appears that barriers to use of prescription footwear may be higher and more unpredictable than initially believed. Previous studies have not considered these risks that participants experience in using prescription footwear and an understanding of them would allow clinicians to address them more appropriately. Once again, this necessitates engaging patients in a shared decision-making process, where they have an opportunity to voice concerns, explore options and make healthcare choices through a facilitative process [48–50].

It is also important to note that this is the first study to shed light on the risk of using prescription footwear in wet weather. This may be due to differences in climates between Singapore and other parts of the world. Therefore, this experience might be unique to patients in Singapore or in countries with similar climate of two distinct monsoon seasons that lead to frequent sudden heavy rainfall [51].

It is evident that the risk of using footwear in wet weather not only has an impact on possible deterioration in foot conditions but also a reduction in quality of life when participants’ ability to go out is limited, and an increased risk of falls with the adoption of dangerous behaviours to keep their shoes dry. Thus, its implications on participants’ social lives and physical health are significant. However, as little was known about this before, the risk of using footwear in wet weather has not been adequately addressed and managed, resulting in greater perceived risk with its use. It is therefore necessary that clinicians in Singapore begin to address appropriate use of prescription footwear in wet weather. This may involve mitigating risky behaviours in the short run but would likely require modification of prescription footwear designs for safer use in Singapore’s climate in the future. More targeted research on necessary features for suitable and acceptable use of footwear in Singapore’s context is necessary to inform the design process and could offer promising improvements in the lives of people with diabetes.

### Personal and social acceptance

Paton and colleagues identified that adaptations of self-perceptions of image and function, along with environmental risk and a pivotal event, such as foot complications, influenced people’s set of personal values regarding footwear choices [31]. Participants in this study, however, go on to suggest that regulation of this set of personal values is not an individuated process but involves aspects of social acceptance. Tenets of social acceptance can either reinforce the process of adapting one’s personal values to accept prescription footwear, or reject it.

This study also showed that social conformability was a necessary facet of social acceptance. Kaiser and colleagues’ study on clothing choices of disabled students might explain this need for social conformability in one’s acceptance of prescription footwear [52]. They suggested that disability only became disruptive when participants appeared different from everyone else. Similarly, the distinct appearance of prescription footwear serves as a revelation of participants’ ‘disabilities’, disrupting an otherwise discreet disease process. A lack of acceptance of prescription footwear, especially in social contexts, further reinforces these differences between participants and others in their social circle. Participants thus reject prescription footwear in an attempt to reduce feelings of inferiority that arose from these distinctions. While improvements in prescription footwear aesthetics have been suggested vaguely before [21, 23, 30, 46], this study supports an emphasis on designing prescription footwear that reflects social norms as previously suggested by Paton and colleagues [31].

### Cultural and social acceptance

Identifying culturally specific health-related norms is crucial in developing models of behaviour change in specific groups [53, 54]. Similarly, socio-cultural norms of footwear use should be taken into account in developing culturally consonant programs supporting its use in Singapore. Adequate use of footwear at home is necessary for individuals to use prescription footwear sufficiently for therapeutic effect [41]. Working against the socio-cultural norm of footwear use at home, it is pertinent that clinicians prescribing footwear provide adequate support by providing an additional pair of prescription footwear for home use, and/or facilitating discussions with individuals and their family about the use of prescription footwear at home.

The extent to which participants accepted the use of prescription footwear appeared to be shaped by elements of acceptance within Singapore’s socio-cultural context. It is revealed as an on-going process of evaluation where social, personal and cultural acceptance contributes to or detracts from the overall sense of acceptance participants felt about using prescription footwear. This interplay of factors regulating footwear acceptance is consistent with the social identity theory that suggests that individuals are born into a society where they derive their sense of self largely from social and cultural situations to which they belong [55]. Therefore, while personal values regarding footwear choice may be unique to individuals, they are never far removed from the influence of social comparison and cultural influence. It is therefore important to consider the socio-cultural impact of using prescription footwear in patients’ lives, as it significantly influences the degree of acceptance participants experience when using it.

### Model influencing Footwêr decision

Within the superordinate themes of security and acceptance, the subthemes of protection and risk, and the interaction of personal, social and cultural acceptance interplay to influence participants’ overall decision to use prescription footwear. These perceptions were revealed not as fixed states that participants arrive at eventually as suggested by Paton and colleagues [31], but were instead situational and fluctuant processes of re-evaluations.

**Fig. 1:**
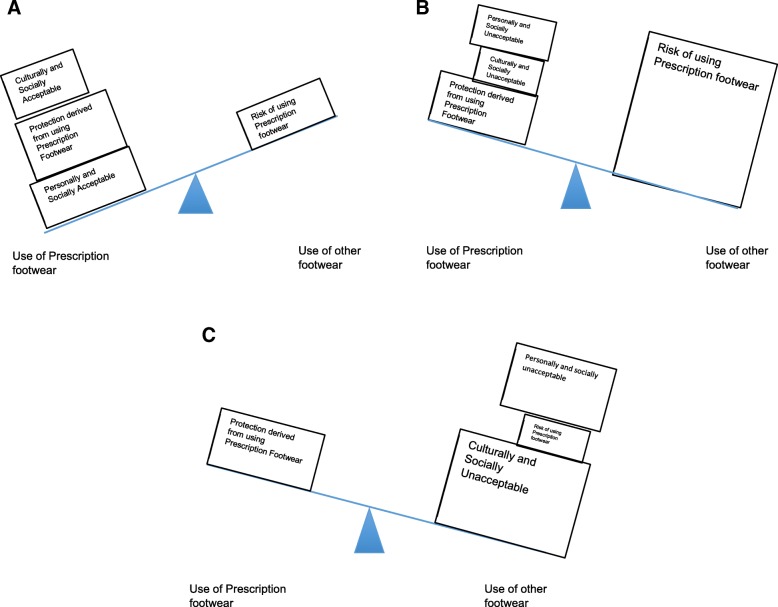
**a**-**c**: Seesaw model illustrating how perceptions of different subthemes affect use of prescription footwear

A modified seesaw model of adherence [28] can be used to illustrate how these themes interact to influence participants’ footwear decisions (Fig. 1).

As depicted by the size of the box, the influence of different subthemes can vary in different circumstances, shifting the decision on the use of prescription footwear either way. While acceptance can occasionally be a personal process, it is never far removed from socio-cultural influences. As such, they are usually on the same side of the seesaw. Figure 1c depicts the typical use of prescription footwear at home in Singapore, where cultural and social norms typically outweigh any other influences on footwear decision, while Fig. 1b depicts the decisions participants face when using prescription footwear in wet weather. This model can be used to understand how participants’ perceptions of factors influencing security and acceptance affect their decision to use prescription footwear. It can also be used to inform clinicians on important influences to address in clinical practice.

### Limitations

This study explores the use of prescription footwear in the unique context of Singapore and may have limited transferability to other populations. Also, participants in this study were mostly males (7 males, 1 female) and additional demographic information on participants was not collected for this study.

It should be acknowledged that female patients who were approached were generally reserved and unwilling to participate in the interview. As female patients tend to report lower satisfaction with footwear appearances [21, 23, 31], it is possible that lower satisfaction with prescription footwear hindered their willingness to participate in the study. As gender differences have been known to influence perspectives and experiences with prescription footwear [23, 31], this study may not be an adequate representation of females’ experiences regarding prescription footwear use in Singapore. Future research designed to specifically explore the experiences of females using prescription footwear in Singapore would be beneficial in allowing a more representative understanding of people’s experience. Use of a sampling framework to recruit a wider variety of eligible participants should also be used in the future.

This paper is also limited in providing insights on the emotional experiences amongst participants using off the shelf prescription footwear. One of the main challenges faced in interviewing participants in this study was helping them understand that they were free to share what they felt, instead of providing ‘answers’ that they have learnt. It seems possible that this may stem from a predominantly paternalistic clinician-patient relationship, where participants were used to passively following their clinicians’ prescriptions. A more collaborative partnership using a shared-decision making process [50] appears necessary to adequately address and explore the emotional complexities that patients experience with the use of prescription footwear in Singapore. This shift is not only necessary on the clinician’s part but also on patients’ willingness to engage in discussion. Further research on how this shift can be made and sustained in Singapore’s context is necessary.

Finally, it should also be acknowledged that participants who signed up to this study are likely to be more confident individuals who have different priorities as compared to more reserved individuals [56]. As such, the findings could be biased towards individuals with higher self-esteem. Future research may consider recruitment methods that encourage the participation of more reserved individuals to gain insight on their possibly varying experiences with prescription footwear.

## Conclusions

This study revealed that participants’ use of prescription footwear in Singapore was influenced by their perception of security and acceptance of its use. A modified seesaw model of this decision-making process (Fig. 1) can be used to illustrate how this takes place. This study also expounds on risks that participants experienced with using prescription footwear in Singapore’s climate while highlighting socio-cultural norms affecting use of prescription footwear in Singapore. While revealing these complexities that participants grappled with, the study also raises several implications for consideration.

The process of this study revealed that participants’ perceptions of security and acceptance were often overlooked in the clinical setting. Underlying participants’ experiences was a common sense of disempowerment where they felt that their opinions about prescription footwear were less valid than those of clinicians’. This questions the importance ascribed to patients’ opinions when prescription of footwear is carried out in Singapore.

The need to constantly weigh pros and cons in footwear decisions is underpinned by a lack of choice [46], where participants are expected to use one type of prescription shoes for all activities. Participants however engaged in a host of activities that required footwear for specific purposes. Evidently, a water-proof option with better safety features appears necessary for use in Singapore’s climate. However, there is also a need for a variety of footwear that reflect mainstream trends for participants’ use in social functions, while preserving therapeutic function and the psychological prompt of comfort. We suggest considering prescription footwear holistically as a means to address emotional, social and physical needs of people with diabetes [57], instead of being solely a medical device.

Providing people with diabetes with access to a range of footwear to meet these needs while fulfilling its therapeutic function may enhance the physical, emotional and psychological security and acceptance they experience with its use, leading to more consistent use of appropriate footwear.

## Abbreviations

DFU: `Diabetic Foot Ulcer
IPA: Interpretative Phenomenological Analysis
VAS: Visual Analogue Scale
